# Evaluation of Four Interventions Using Behavioral Economics Insights to Increase Demand for Voluntary Medical Male Circumcision in South Africa Through the MoyaApp: A Quasi-Experimental Study

**DOI:** 10.1097/QAI.0000000000003504

**Published:** 2024-08-09

**Authors:** Preethi Mistri, Silviu Tomescu, Simamkele Bokolo, Alexandra De Nooy, Pedro T. Pisa, Skye Grove, Laura Schmucker, Candice Chetty-Makkan, Lawrence Long, Alison Buttenheim, Brendan Maughan-Brown

**Affiliations:** a Faculty of Health Sciences, Health Economics and Epidemiology Research Office, University of the Witwatersrand, Johannesburg, South Africa; b Right to Care, Department of Strategic Information, Centurion, South Africa;; c Department of Human Nutrition and Dietetics, University of Pretoria, Pretoria, South Africa;; d Right to Care, Department of Social and Behaviour Change Communication, Centurion, South Africa;; e Department of Medical Ethics and Health Policy, Perelman School of Medicine, University of Pennsylvania, Philadelphia, PA;; f Department of Global Health, Boston University School of Public Health, Boston, MA;; g Department of Family and Community Health, School of Nursing, University of Pennsylvania, Philadelphia, PA; and; h Southern Africa Labour and Development Research Unit, University of Cape Town, Cape Town, South Africa.

**Keywords:** HIV prevention, male circumcision, message framing, behavioral economics, foot-in-the-door, social norms

## Abstract

**Background:**

While voluntary medical male circumcision (VMMC) reduces the risk of HIV transmission by 60%, circumcision coverage falls short of the UNAIDS 90% VMMC target. We investigated whether behaviorally informed message framing increased demand for VMMC.

**Setting:**

Adult users of the MoyaApp, a data-free application in South Africa, who viewed a form designed to generate interest in VMMC from August 2022 to November 2022.

**Methods:**

A quasi-experimental study was conducted to evaluate 4 MoyaApp VMMC intervention forms against the standard-of-care (SOC) form. All forms enabled users to provide contact details for follow-up engagement by a call center. The primary outcome was the proportion of forms submitted. Secondary outcomes included successful contact with the user, VMMC bookings/referrals, and confirmed circumcision. Multivariable ordinary least-squares regression was used for the analysis.

**Results:**

Of 118,337 MoyaApp VMMC form viewers, 6% submitted a form. foot-in-the-door form viewers were more likely (+1.3 percentage points, *P* < 0.01) to submit a form compared with the SOC group (6.3%). Active Choice (−1.1 percentage points, *P* < 0.01) and Reserved for You (−0.05 percentage points, *P* < 0.05) form viewers were less likely to submit a form compared with SOC form. Users submitting the foot-in-the-door form were less likely to be booked/referred compared with those using the SOC form (−5 percentage points, *P* < 0.05). There were no differences between the intervention and SOC forms for successful contact and circumcisions.

**Conclusions:**

Message framing using behavioral insights was able to nudge men to engage with VMMC services. However, more work is needed to understand how to convert initial interest into bookings and circumcisions.

## Abstract 2

Supplemental Digital Content is Available in the Text.

## INTRODUCTION

Voluntary medical male circumcision (VMMC) is effective in reducing the risk of HIV transmission (women to men by approximately 60%^1^ and men to men by approximately 23%^2^) and offers lifelong risk reduction for HIV transmission.^3^ Between 2008 and 2019, 26.8 million men and boys were circumcised in sub-Saharan Africa with an estimated 340,000 new HIV infections averted.^4^ The WHO and UNAIDS recommend VMMC as a core HIV-prevention strategy and set a 90% VMMC target coverage in high-priority countries, including South Africa.^5^ While more than 4.4 million men in South Africa have been circumcised, the country still falls considerably short of the 90% target with only 62.5% of men between 15 and 49 years circumcised by 2022.^6^

Barriers to VMMC include fear of pain, concerns about postprocedure abstinence and missed work, stigma around recommended precircumcision HIV testing, perceived low HIV risk, and the belief that VMMC is not appropriate for older men.^7,8^ In addition, men are less likely to engage in HIV health services than women, which may be exacerbated by concerns regarding confidentiality, perceptions of compromised masculinity, and stigma.^1,9^

Demand creation strategies have been central to the scale-up of VMMC in priority countries.^8,10,11^ Behavior change communication strategies and other innovative approaches have been used to improve knowledge, awareness, and engagement in medical circumcision.^8,12^ These include tailoring messages to men's stage of behavior change (ie, behavior change can be a multistage process with varying levels of awareness, readiness, intentions, etc, at each stage), including benefits other than just HIV prevention in messaging, promoting positive social norms, and addressing individual and environmental barriers.^8,12^

In general, health decisions may be influenced based on how they are framed or presented^13^ and also by psychological, behavioral, emotional, social, and contextual factors.^14^ Messages can be constructed to address specific behavioral barriers to overcome a potential intention-action gap or increase intention.^12,15^ We used message framing leveraging behavioral economics principles to increase demand for VMMC in South Africa.

## METHODS

### Study Design and Population

This was a 5-arm quasi-experimental, nonrandomized group study conducted from August 8, 2022, to November 21, 2022, across all of South Africa's 9 provinces. The target sample included adults (≥18 years old) registered on the MoyaApp who accessed a VMMC form during the study period.

The MoyaApp is a data-free mobile device application (app) that offers its users a range of data-free content including engagement with services and access to information like the weather and sports.^16^ It is available on the 4 main mobile network providers in South Africa that together offer national coverage.^16^ There are approximately 4 million daily and 6.5 million monthly MoyaApp users,^16^ indicating use by more than 15% of adult South Africans.^17^ A VMMC icon in the MoyaApp health section directs individuals to a VMMC form where they can submit contact details as an expression of interest for a call back by program counselors. The VMMC form on the MoyaApp and related VMMC demand generation activities are implemented by Right to Care (RTC), a healthcare organization supporting HIV prevention and treatment programs in South Africa.

The VMMC form is created in Microsoft Forms and manually uploaded to the MoyaApp platform, which can only host one form at any given time. This study evaluated 4 new intervention forms against the standard-of-care (SOC) form (5 forms overall). The forms were allocated across 45 blocks of time in the 15-week study period, with forms changed at approximately 8 am every Monday, Wednesday, and Friday—a rotation cadence that was operationally feasible within the VMMC program. Forms were allocated consecutively to time blocks in a predetermined randomized sequence repeated 9 times across the study period to account for potential temporal influences on app usage and circumcision demand (see Table S1, Supplemental Digital Content, http://links.lww.com/QAI/C336). Access to the forms was standard across all the forms, and all the forms were in English.

Submission of a form by a MoyaApp user generated an automated email to the call center, triggering counselors to contact individuals telephonically. Counselors at the call center provided information on VMMC, answered questions, and then either scheduled an appointment for the procedure or referred interested clients to other service providers. Telephonic engagement with clients by counselors followed standard procedures, was not scripted, and was consistent across study groups. Counselors were not blinded to study group allocation. Counselors made multiple attempts to reach clients using both primary and secondary (if provided) contact numbers.

### Control Group: SOC Form

The SOC form contained a banner identifying the medical circumcision program, an image, and brief (<60 words) text providing general information and health benefits of VMMC. User-completed fields included name, surname, contact number(s), and age category (≥18 years old or under 18 years).

See Table 1 for a detailed description of the SOC and intervention forms.

**TABLE 1.** Form Title and Messaging

Standard of Care	Foot-in-the-Door	Stand Proud	Reserved for You	Active Choice
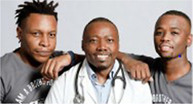	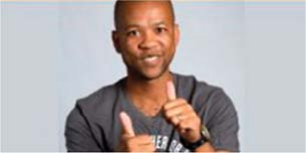	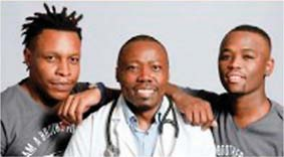	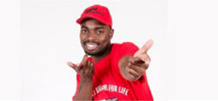	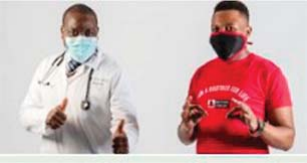
If you are over 15, sign up here for safe, FREE medical circumcision today. You are never too old to circumcise. Studies show that 78% of women prefer circumcised men. Stand proud and protect yourself and your partner. Sign up here for safe, FREE medical circumcision today. It lowers your risks of HIV and STIs and improves your hygiene.	Say YES to talking to a counselor! Are you interested in medical circumcision and would like to know more? We are ready to tell you more about safe, FREE medical circumcision. Sign up here and a counselor will give you a call to answer your questions.	Stand Proud and go for medical circumcision Every day more than 100 men like you sign up here to make a booking for circumcision. They know they can book a safe, FREE medical circumcision appointment with a counselor. Stand proud with your brothers!	We can reserve a medical circumcision appointment just for you. Sign up to claim it today! Do you want to set up an appointment for medical circumcision? One of our counselors can call you and keep one of our limited appointments just for you. Don't wait—sign up today to claim personal attention and a safe, FREE medical circumcision.	Medical Circumcision: You choose! Get circumcised and protect not only yourself but your partner too. Choose your path below to get your questions answered and get your medical circumcision booked. Choose your path: Yes! I am ready—Call me to book a safe, FREE medical circumcision appointment. Yes! I have a question—Call me to talk through my questions about medical circumcision.
**Behavioral Economics Principles**				
Provided information.	Created the opportunity for smaller, more manageable steps to be taken before the larger decision of committing to a circumcision.	Utilized social norms to leverage the male identity, creating a sense of belonging.	Leveraged loss aversion, the endowment effect, and exclusivity to create a sense of ownership and to motivate users to follow through and not lose the opportunity.	Offered users agency to proactively choose which “path” to take, rather than a default, reducing pressure to commit to a circumcision.

### Interventions

The intervention forms were co-designed with RTC VMMC Program Strategic Information, Data, Technical and Operational teams who provided insights on barriers to VMMC services. Behavioral economics principles leveraged included social norms, loss aversion, the endowment effect, exclusivity, the foot-in-the-door persuasion technique, and Active Choice. Message framing made the steps toward VMMC feel easy and low-cost.

Six prototype forms were created based on the experience of the RTC team engaging with clients and common barriers to VMMC reported by clients, and were refined through iterative rounds of feedback from the RTC team. The final 4 forms perceived to be most likely to increase demand for VMMC were selected jointly with RTC for evaluation. Intervention forms included the same fields for contact details as the SOC form.

#### Intervention Form 1: Foot-in-the-Door

The foot-in-the-door persuasion technique in which individuals are asked to take a small initial step toward a big decision^18–20^ was used to frame form submission and telephonic engagement with a counselor as a small, manageable step before the larger decision to commit to VMMC.

#### Intervention Form 2: Stand Proud

The Stand Proud form was designed on the premise that individual behavior is greatly influenced by what people see or hear of others doing. Social norms—the informal rules of beliefs, attitudes, and behaviors that are considered acceptable in a particular society or social group^21^—have been leveraged to influence a range of behaviors.^22,23^

#### Intervention Form 3: Reserved for You

Framing a service or product as “reserved for you” creates a sense of exclusivity, and amplifies the perceived value of the offer.^24^ These so-called “ownership prompts” may also invoke loss aversion (an individual's tendency to prefer avoiding losses to acquiring similar gains) and the endowment effect^25^ personalizing the appointment booking process and addressing the perceived inconvenience of VMMC services.

#### Intervention Form 4: Active Choice

This form provided users agency to choose a path, and to reduce pressure to commit to VMMC by requesting them to proactively make a choice rather than having a default choice already selected.^26^ The decision architecture catered to people who may have already decided to go ahead with circumcision and were ready to make a booking, as well as those people who still had questions about VMMC and needed further engagement and information before making the decision.

### Data and Outcomes

Routine administrative data were used for this study, with no primary data collection. The primary study outcome was the proportion of MoyaApp users viewing a VMMC submission form who then submitted the form. MoyaApp provided data on the number of MoyaApp users ≥18 years (based on stated age in Moya registrations) who viewed a VMMC form in each of the 45 study blocks (ie, between the time a form was uploaded to when it was changed), which formed the denominator. The RTC VMMC program collected data on form submissions using Microsoft Forms.

Three binary variables were created as secondary outcomes that were based on the key steps that individuals took along the VMMC journey: 1) *Contacted* by the call center captured whether the call center was able to speak with someone telephonically using the contact details provided on the submitted VMMC form; 2) *Booked/referred* for a VMMC appointment identified individuals whom the call center agent spoke with and who were either booked directly for a VMMC appointment or referred to another service provider (which is the protocol for clients residing outside of regions where RTC operates); and 3) *Circumcised* identified individuals who were recorded in the clinic system as having attended their VMMC appointment and been circumcised.

Data from the RTC VMMC program were used for the secondary outcomes. Data on telephone contact by the call center and bookings/referrals were extracted from RTC call center databases. Data for attending a clinic visit for the VMMC procedure was routinely linked to RTC VMMC program data (using national identity numbers) from the RightMax Lynx electronic clinic data system, a cloud-based database used by RTC at RTC-supported facilities. Data on factors associated with successful booking were not routinely collected within the RTC administrative databases. A unique study identification number (ID) was created for every individual who submitted a form, with repeat form submissions and call center outcomes identified by cell phone number and assigned to the relevant ID.

### Statistical Analysis

χ^2^ tests assessed the difference in proportions between the SOC form and each of the intervention forms for each outcome. For ease of interpretation, linear probability models (ordinary least-squares regression) were used to estimate the effect of each intervention form relative to the SOC, with adjustments to account for the clustering of observations within each of the 45 study blocks, and controlling for potential study week and day of week (Monday, Wednesday, or Friday) effects.^27^ For robustness, we also estimated logistic regression models. Analyses were conducted in Stata V.17.^28^

#### Form Submissions (Primary Outcome)

Data were excluded (n = 26) when a form was submitted during the period in which the form was being changed from one study group to another, and it was not possible to know which form was viewed by the individual. The unit of analysis was the form (ie, if someone submitted on 2 separate forms then the submission was counted for each separate form).

#### Call Center Contact, VMMC Booking, and Circumcision (Secondary Outcomes)

The impact of the intervention on the secondary outcomes was assessed among the subsample of individuals who both submitted a form and were included in the list of individuals for subsequent contact.

The analysis of the intervention effects on the secondary outcomes was designed as an individual-level analysis (ie, the number of individuals in these analyses is smaller than the total number of forms submitted as some individuals submitted multiple forms). Individuals were assigned to a study group based on the first form they submitted, with the rationale that exposure to the first form could influence all subsequent decisions. Additional regression analysis was conducted for the secondary outcomes to assess for robustness to the inclusion of an additional binary control variable which captured whether an individual submitted multiple forms (=1) or not (=0). It is possible that the call center could have made more attempts to contact these individuals if the details submitted were not recognized as duplicates.

An administrative data system error resulted in a subset of individuals who submitted a form (2498/6652) not being included in the contact list (ie, no contact attempts were made based on these forms). These individuals were excluded from the analysis. Logistic regression analyses (results not shown) found that individuals who submitted the SOC form were more likely to be excluded from the contact list than individuals who submitted study forms.

### Ethical Considerations and Trial Registration

This study was approved by the Human Research Ethics Committee (Medical) of the University of the Witwatersrand, the University of Pennsylvania, and Boston University institutional review boards. A waiver of informed consent was granted for this study which utilized anonymized administrative data for MoyaApp users ≥18 years.

This study was registered with the Pan-African Clinical Trials Registry PACTR202112699416418 and the South African Clinical Trials Registry DOH-27-062,022-7811.

## RESULTS

**FIGURE 1.**
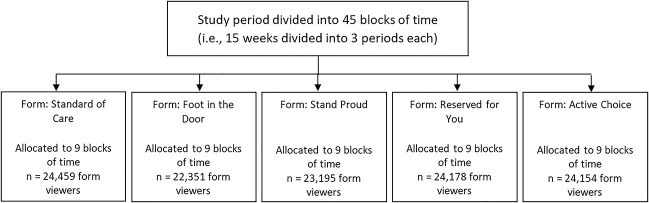
Flow of study participants for MoyaApp voluntary medical male circumcision form viewers, aged 18 years and older.

There were 118,337 MoyaApp VMMC form viewers ≥18 years during the study period. The number of form viewers included in the primary analysis was 24,459 (20.7%) for SOC, 22,351 (18.9%) for foot-in-the-door, 23,195 (19.6%) for Stand Proud, 24,178 (20.4%) for Reserved for You, and 24,154 (20.4%) for Active Choice (Fig. 1).

### Form Submissions (Primary Analysis)

**FIGURE 2.**
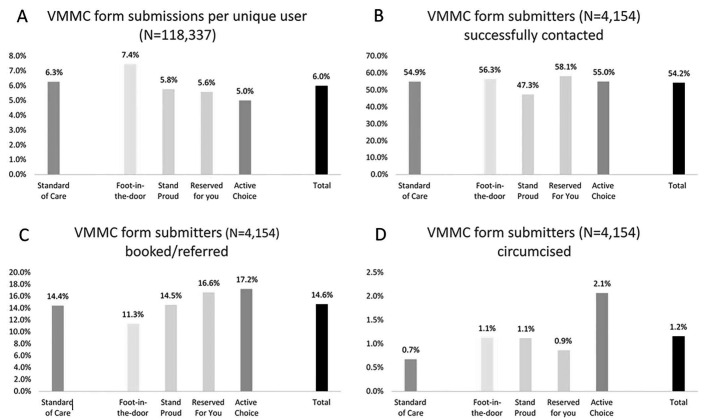
Proportion of MoyaApp users submitting forms and proportions of form submitters successfully contacted, booked/referred and circumcised.

Of 118,337 form viewers across all study blocks, 7089 (6%) submitted a VMMC form with 1532 (6.3%) submitting on the SOC form. A higher proportion submitted the foot-in-the-door form (7.4%, n = 1,664) than the SOC form (*P* < 0.01). The proportion submitting in all other forms was lower than the SOC form: Stand Proud (5.8%, n = 1,336, *P* < 0.05), Reserved for You (5.6%, n = 1,348, *P* < 0.01), and Active Choice (5.0%, n = 1,209, *P* < 0.01; Fig. 2A).

Ordinary least-squares regression results, adjusted for potential confounders (Table 2, model 2), showed that viewers of the foot-in-the-door form were more likely (+1.3 percentage points, *P* < 0.01) to submit than those who viewed the SOC form. By contrast, compared with the SOC form, submissions were lower on the Active Choice (−1.1 percentage points, *P* < 0.01) and Reserved for You forms (−0.05 percentage points, *P* < 0.05). Associations with day of the week and study week number controls can be seen in Table S2, Supplemental Digital Content, http://links.lww.com/QAI/C336. Results were substantively similar in logistic regression models (see Table S3, Supplemental Digital Content, http://links.lww.com/QAI/C336).

**TABLE 2.** Ordinary Least-Squares Regression Models Assessing the Association Between VMMC Forms Submitted Across 5 Arms by Adults Who Viewed the Forms During August and November 2022 in South Africa (N = 118,337)

	N	% Submissions	Submitted Forms	
			Unadjusted (Model 1), β (95% CI)	Adjusted (Model 2), β (95% CI)
Study groups				
SOC (ref)	24,459	6.3		
Foot-in-the--Door	22,351	7.4	0.012 (0.004 to 0.019)*	0.013 (0.009 to 0.017)*
Stand Proud	23,195	5.8	−0.005 (−0.012 to 0.002)	−0.002 (−0.008 to 0.003)
Reserved for You	24,178	5.6	−0.007 (−0.014 to −0.000)**	−0.005 (−0.009 to −0.001)***
Active Choice	24,154	5	−0.013 (−0.019 to −0.006)*	−0.011 (−0.017 to −0.006)*
Control variables included			No	Yes
Observations			118,337	118,337
R^2^			0.001	0.002

Control variables were study week and day of week (Monday, Wednesday, or Friday).

* *P* < 0.01.

** *P* < 0.1.

*** *P* < 0.05.

### Call Center Contact, VMMC Booking, and Circumcision (Secondary Analysis)

The secondary analysis included the subsample of 4154 individuals who submitted a form and their form was included in the list of individuals to be contacted by the call center. Additional analyses (see Table S4, Supplemental Digital Content, http://links.lww.com/QAI/C336) showed that the SOC form was excluded from the contact list more frequently than the intervention forms.

### Contacted

Of the adult users submitting a form and who were on the contact list, 54.2% (n = 2,253) were successfully contacted. More users were contacted after submitting a Reserved for You (58.1%, n = 472) and foot-in-the-door (56.3%, n = 551) form compared with SOC form (54.9%, n = 408), Active Choice about the same (55.0%, n = 399), and Stand Proud fewer (47.3%, n = 423; Fig. 2B).

Regression results (Table 3, model 2) showed that the difference in successful contacts between the SOC and any of the intervention forms was not statistically different after accounting for clustering and adjusting for potential confounders. Full model results for all secondary analyses are provided in Table S5, Supplemental Digital Content, http://links.lww.com/QAI/C336.

**TABLE 3.** Ordinary Least-Squares Regression Models of the Impact of the Study Forms on Secondary Study Outcomes: Successful Contact by the Call Center, Booking/Referral Complete, and Circumcision Complete

	N	% Contacted	Contacted		% Booked/Referred	Booked/Referred		% Circumcised	Circumcised	
			Unadjusted (Model 1), β (95% CI)	Adjusted (Model 2), β (95% CI)		Unadjusted (Model 3), β (95% CI)	Adjusted (Model 4), β (95% CI)		Unadjusted (Model 5), β (95% CI)	Adjusted (Model 6), β (95% CI)
Study groups										
SOC (ref)	743	54.9			14.4			0.7		
Foot-in-the-Door	978	56.3	0.014 (−0.053 to 0.081)	−0.016 (−0.068 to 0.037)	11.3	−0.031 (−0.073 to 0.012)	−0.050 (−0.092 to −0.009)*	1.1	0.005 (−0.004 to 0.013)	0.002 (−0.007 to 0.011)
Stand Proud	895	47.3	−0.076 (−0.145 to −0.008)*	−0.038 (−0.091 to 0.016)	14.5	0.001 (−0.041 to 0.043)	−0.031 (−0.081 to 0.018)	1.1	0.004 (−0.005 to 0.014)	0 (−0.012 to 0.011)
Reserved for You	812	58.1	0.032 (−0.048 to 0.112)	0.040 (−0.007 to 0.087)**	16.6	0.022 (−0.029 to 0.073)	0.01 (−0.032 to 0.053)	0.9	0.002 (−0.007 to 0.011)	0.001 (−0.008 to 0.011)
Active Choice	726	55.0	0 (−0.066 to 0.067)	0.036 (−0.021 to 0.093)	17.2	0.028 (−0.016 to 0.073)	0.011 (−0.038 to 0.059)	2.1	0.014 (0.000 to 0.027)*	0.012 (−0.000 to 0.025)†
Control variables included			No	Yes		No	Yes		No	Yes
Observations			4154	4154		4154	4154		4154	4154
R^2^			0.006	0.021		0.004	0.009		0.002	0.005

Control variables were study week and day of week (Monday, Wednesday, or Friday).

* *P* < 0.05.

** *P* < 0.1.

### Booked/Referred

Among form submitters, 14.6% (n = 608) were booked or referred for VMMC. Compared with SOC forms (14.4%, n = 107), more bookings were made among individuals who submitted on Active Choice (17.2%, n = 125), Reserved for You (16.6%, n = 135), and Stand Proud (14.5%, n = 130) forms, and fewer bookings/referrals were made among those who submitted a foot-in-the-door form (11.3%, n = 111) (Fig. 2C). The adjusted regression analysis (Table 3, model 4) found that users submitting on the foot-in-the-door form were *less* likely to be booked/referred compared with SOC form (−5 percentage points, *P* < 0.05). None of the other relationships were different at *P* < 0.05.

### Circumcised

A total of 48 individuals (1.2%, n = 48) in our subsample were recorded in clinic records as having been circumcised. Compared with the SOC form, a greater proportion submitting on all other forms were circumcised (Fig. 2D), but differences were not statistically significant in the adjusted regression analysis (Table 3, model 6). The regression results for the secondary analysis were substantively similar in logistic regression models (see Table S6, Supplemental Digital Content, http://links.lww.com/QAI/C336) and with the inclusion of a control variable to account for someone submitting multiple forms (see Table S7, Supplemental Digital Content, http://links.lww.com/QAI/C336).

## DISCUSSION

This study found that messages framed using behavioral economics principles could encourage more MoyaApp users to submit their details for VMMC. Users viewing the form that leveraged the foot-in-the-door technique were significantly more likely to submit a form compared with those viewing the SOC form. Those who viewed the Active Choice and Reserved for You forms were significantly less likely to submit a form for further engagement. Studies utilizing the foot-in-the-door technique in HIV prevention in low- and middle-income countries are limited. One study in the United States showed that creating opportunities for participants to engage in HIV-prevention activities ranging from lighter or less time-consuming activities to progressively heavier or more time-consuming activities (the foot-in-the-door concept) was effective: perusing an HIV-prevention brochure increased watching a 10-minute video on HIV prevention, and watching the video increased likelihood of engaging in a counseling session.^19^

Research in other contexts and experimental conditions have shown mixed results and indicate that the foot-in-the-door effect may be influenced by various psychological processes (eg, self-perception and intention) and that the strength of these processes can either enhance or reduce the effect.^29,30^ Influencing attitudes, social norms, and perceived behavioral control can strengthen the intention to carry out health behaviors,^15^ in this case, acting on the intention to undergo medical circumcision.

While the foot-in-the-door form did better at encouraging form submissions, it did worse in terms of successful bookings/referrals for an appointment. By contrast, messaging based on Reserved for You and Active Choice principles performed marginally better than the SOC form on bookings for VMMC (although differences were not statistically significant) despite having generated relatively fewer initial form submissions. Circumcision outcomes for MoyaApp users submitting VMMC forms were not different between the intervention and SOC forms.

It is possible that the foot-in-the-door technique encouraged more submissions because it lowered the sense of initial commitment. However, people who submitted the foot-in-the-door form may have also been less interested in VMMC overall than those submitting other forms. It is also possible that an increase in submissions from a successful form resulted in increased pressure on the call center due to greater volumes of people to contact. It is not known whether the additional volume of pending calls could have influenced the time spent, quality, and the extent to which questions were addressed in discussions the call center agents had with potential clients. Since more bookings were made on the Stand Proud, Reserved for You, and Active Choice forms, combining aspects of these forms with foot-in-the-door may have the potential for an enhanced effect and should be investigated further.

Following form submission, call center engagement was standardized and did not consider the specific messaging per the MoyaApp VMMC form. It would be important to explore whether an intervention at the stage of call center contact that continues the behaviorally informed dialogue started in the form, could be effective in motivating men to overcome the intention-action gap to being circumcised. Behaviorally informed scripts could be used by call center agents to align discussions with the behavioral economics principles applied in the MoyaApp VMMC form. Such scripts should consider the stage of readiness for circumcision and specific questions that have been raised before. A VMMC framework using market research and a person-centered approach in Zambia and Zimbabwe identified 3 stages (relate, anticipate, relieve) in understanding steps toward medical circumcision.^12^ Each stage included psychological processes that men may grapple with when following through on the intention to get circumcised.^12^ The framework could be used to enhance the intervention forms and inform scripts that call center counselors use to encourage acting on the intention to circumcise.^12^

The results of this study should be interpreted alongside its limitations. Since only one VMMC form was viewable in the MoyaApp at a time, it was not possible to randomize users to study arms. Furthermore, there was a deviation in the predetermined sequence of the forms appearing in the MoyaApp, resulting in the same form appearing on 2 consecutive Fridays at 2 points in this study. While this might have impacted form submissions, any bias was likely mitigated by controlling for temporal influences on form submissions in the regression analyses. As noted previously, because of a data system error a subset of MoyaApp users who submitted forms were not attempted to be contacted by the call center. Submissions on the SOC form were more likely to be excluded from follow-up contact by the call center than submissions on each of the intervention forms. It is unclear whether this might have biased results.

This study was limited to data from MoyaApp users and VMMC program data for people ≥18 years. A recent study indicated that younger men (below 20 years old) often access medical circumcision more than older men (20–34 years).^31^ Younger men are an important target population for medical circumcision, and it is important to assess the impact of message framing among these individuals. In addition, all forms were in English, which may have limited the study data to users able to engage in English.

### Conclusions

Message framing using behavioral economics principles can be used in the HIV context to nudge men to engage with health services. The foot-in-the-door intervention was effective in increasing interest in medical circumcision. However, more work is needed on motivating the continuation of this interest and conversion to acting on the intention to VMMC downstream of the initial interest. Incorporating behavioral economics strategies into targeted interventions that are less expensive, scalable, and person-centered can be effective in motivating engagement in care. The use of technology provides a low-cost and convenient platform to implement interventions to encourage the uptake of health services. Future research on digital message framing interventions to increase VMMC should include younger men, who may engage with technology differently and are more likely than older men to get circumcised. Learnings from this work could also be applied and evaluated in other areas of HIV prevention and treatment.

## Supplementary Material

SUPPLEMENTARY MATERIAL
